# Artificial Intelligence vs. Statistical Modeling and Optimization of Continuous Bead Milling Process for Bacterial Cell Lysis

**DOI:** 10.3389/fmicb.2016.01852

**Published:** 2016-11-22

**Authors:** Shafiul Haque, Saif Khan, Mohd Wahid, Sajad A. Dar, Nipunjot Soni, Raju K. Mandal, Vineeta Singh, Dileep Tiwari, Mohtashim Lohani, Mohammed Y. Areeshi, Thavendran Govender, Hendrik G. Kruger, Arshad Jawed

**Affiliations:** ^1^Department of Biosciences, Jamia Millia Islamia (A Central University)New Delhi, India; ^2^Research and Scientific Studies Unit, College of Nursing and Allied Health Sciences, Jazan UniversityJazan, Saudi Arabia; ^3^Department of Clinical Nutrition, College of Applied Medical Sciences, University of Ha’ilHa’il, Saudi Arabia; ^4^Centre for Interdisciplinary Research in Basic Sciences, Jamia Millia Islamia (A Central University)New Delhi, India; ^5^The University College of Medical Sciences and Guru Teg Bahadur Hospital (University of Delhi)New Delhi, India; ^6^Department of Biotechnology, Khalsa CollegePatiala, India; ^7^Microbiology Division, Council of Scientific and Industrial Research – Central Drug Research InstituteLucknow, India; ^8^Catalysis and Peptide Research Unit, School of Health Sciences, University of KwaZulu-NatalDurban, South Africa; ^9^RFCL LimitedNew Delhi, India

**Keywords:** cell lysis, response surface methodology, central composite design, artificial neural network, genetic algorithm, cholesterol oxidase

## Abstract

For a commercially viable recombinant intracellular protein production process, efficient cell lysis and protein release is a major bottleneck. The recovery of recombinant protein, cholesterol oxidase (COD) was studied in a continuous bead milling process. A full factorial response surface methodology (RSM) design was employed and compared to artificial neural networks coupled with genetic algorithm (ANN-GA). Significant process variables, cell slurry feed rate (A), bead load (B), cell load (C), and run time (D), were investigated and optimized for maximizing COD recovery. RSM predicted an optimum of feed rate of 310.73 mL/h, bead loading of 79.9% (v/v), cell loading OD_600__nm_ of 74, and run time of 29.9 min with a recovery of ~3.2 g/L. ANN-GA predicted a maximum COD recovery of ~3.5 g/L at an optimum feed rate (mL/h): 258.08, bead loading (%, v/v): 80%, cell loading (OD_600__nm_): 73.99, and run time of 32 min. An overall 3.7-fold increase in productivity is obtained when compared to a batch process. Optimization and comparison of statistical vs. artificial intelligence techniques in continuous bead milling process has been attempted for the very first time in our study. We were able to successfully represent the complex non-linear multivariable dependence of enzyme recovery on bead milling parameters. The quadratic second order response functions are not flexible enough to represent such complex non-linear dependence. ANN being a summation function of multiple layers are capable to represent complex non-linear dependence of variables in this case; enzyme recovery as a function of bead milling parameters. Since GA can even optimize discontinuous functions present study cites a perfect example of using machine learning (ANN) in combination with evolutionary optimization (GA) for representing undefined biological functions which is the case for common industrial processes involving biological moieties.

**GRAPHICAL ABSTRACT:**
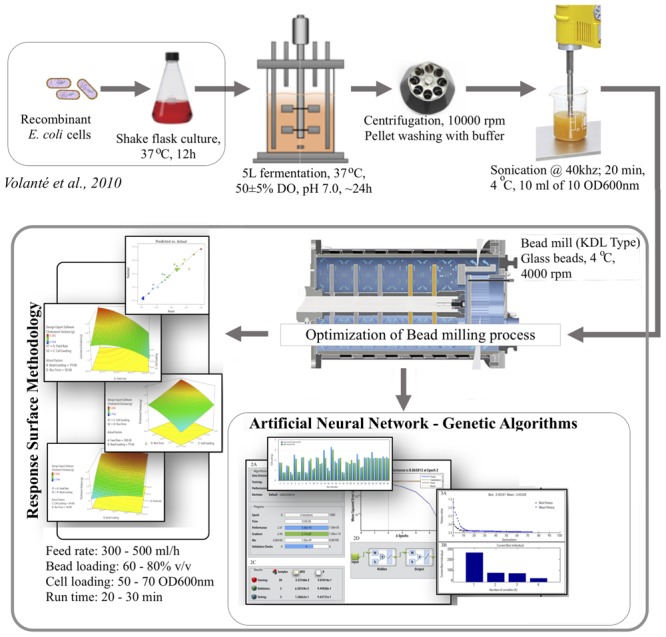
**Schematic representation of modeling and optimization of bacterial cell lysis process by using statistical and artificial intelligence technique for COD recovery**.

## Introduction

Intracellular production of recombinant proteins in *Escherichia coli* has been firmly established a method of choice due to its faster growth, well-characterized genetics, higher protein expression and yield (Huang et al., 2000). Apart from the benefits, production of the recombinant protein suffers a number of bottlenecks, right from gene cloning to its expression and its recovery in the pure and active state (Rosano and Ceccarelli, 2014). Out of all the unit operations, cell lysis and release of intracellular product remains a major challenge toward achieving higher productivity (Shokri et al., 2003). Therefore, modeling and optimization of the cell lysis process becomes critical for achieving higher product recoveries. At laboratory level, the purity of the protein is a prime concern while at commercial scale purity along with yield is critical. Cell disruption methods are normally selected on the basis of the intentional use and nature of the protein product to be recovered, e.g., its thermal-/pH-stability, half-life, activity, etc. (Middleberg, 1995). Unfortunately, the commonly used cell lysis methods including chemical, physical, or enzymatic (Hughes et al., 1971; Fish and Lilly, 1984; Morein et al., 1994; Byreddy et al., 2015) prove inefficient when attempted at larger scale. Alternatively, mechanical methods such as high-pressure homogenization, bead milling are milder, result in lower protein degradation, non-selective, have comparable efficiency and scalable to pilot and process scale (Middleberg, 1995). Bead milling is generally preferred at larger scale owing to its easy control and operation under batch or continuous mode and capability to process volumes of highly concentrated cell slurry (Middleberg, 1995). Scaled-down bead mills, e.g., Dynomill^®^ (WAG, Switzerland) efficiently simulates large-scale bead mills, but it is not routinely used in laboratories due to its relatively high cost. Bead-milling process generates a lot of heat that is directly proportional to the amount of beads present in the grinding chamber, agitation speed, and grinding time. Therefore, cell lysis optimizations need to focus on shortening the run time and minimize the amount of beads loaded, without compromising or even increasing the productivity of the process.

Process optimization often involve simple, conventional one-factor-at-a-time (OFAT) approach. The process suffers major drawbacks of being time consuming, inaccurate, with pseudo-optimum predictions and fail to study the interactions of different process variables affecting the final yield (Wahid and Nadir, 2013). Whereas, statistical methods, i.e., response surface methodology (RSM), and mathematical modeling *via* artificial neural networks (ANNs) methods are accurate, require less experimental number of runs, approach the correct optima; these are more accurate and account for the interaction of process variables involved (Dubey et al., 2011). RSM has been successfully applied to various biotechnological processes in the past (Alshammari et al., 2015) including high throughput chemical lysis of *E. coli* (Listwan et al., 2010) and high throughput development and optimization of efficient cell disruption methods (Glauche et al., 2016). When the number of variables increase and interactions become too complex, RSM fails to describe the object functions accurately. On the contrary, ANN successfully addresses and overcomes the limitations of RSM. ANNs are effective in handling data with noise, mimic biological neural networks, perform better than statistical/regression based models and accurately predict and model highly non-linear and complex biological processes (Khan et al., 2011). The ANN training function Levenberg–Marquardt (LM) is an established curve fitting algorithm for multivariable non-linear least square problems. During ANN training the LM iterator continuously modifies the layer waits and biases (function constants) so that the network output approaches minimum least square difference between the training values and network predictions. LM algorithm behaves somewhere between two learning functions: Steepest Descent and Gauss–Newton. LM requires an initial starting point provided as initial layer weights and biases (for ANN learning). If the current solution is far from correct then LM primarily behaves as steepest descent where it may be slower but determined to reach minima (Moré, 1978, Lourakis, 2005). The precision of the optimized statistical/mathematical models can be increased further by using complimenting tools, such as genetic algorithms (GA), Nelder–Mead complex, etc. GA lays its foundation on Darwin’s principle of genetic evolution (Haider et al., 2008). It intrinsically employs genetic operators like mutation, crossover, and selection to reach at the optimum conditions. It also possesses a unique advantage, as it can be used in the optimization processes where the model to be employed is incomplete or suffers gaps in design execution (Tripathi et al., 2012). In terms of its application, the variables are represented as genomes or chromosomes and the factors to be optimized, i.e., the levels of these variables are portrayed to be genes. Chromosomes that offer higher productivity are selected and replicated proportionally to the achieved productivity. The individual process variables are selected entirely in a random fashion from the population and used to produce the next generation. The population thus evolves over successive generations toward an optimal solution (Haider et al., 2008).

In our previous study, we have reported modeling and optimization of continuous bead milling process for efficient bacterial cell lysis for the production of cholesterol oxidase (COD) using RSM (Haque et al., 2016). In continuation to the previous report, here in this study COD was considered as a prototypical enzyme for the modeling and optimization of bacterial cell lysis employing RSM vs. ANN methodology. COD catalyzes the conversion of cholesterol into 4-cholesten-3-one in the presence of O_2_ and isomerization of 4-cholesten-3-one into 4-3-ketosteroid (Goswami et al., 2013). COD has been receiving a lot of attention due to its broad application in clinical laboratories for quantification of serum cholesterol levels, as a biocatalyst for steroid production, its implication in bacterial and viral manifestations, development of biosensors, as larvicidal and insecticidal protein and as signaling protein in the biosynthesis of polyene macrolide pimaricin in some *Streptomyces* sp. (Mendes et al., 2007). An in-house economic analysis was also performed for assessing the feasibility of the process and quantify the efficiency and productivity of the developed model, similar to our previous report (Haque et al., 2016).

Overall, in this study, artificial intelligence techniques, ANN-GA was applied and compared with statistical design of experiments (DOE) for the modeling and optimization of bacterial cell lysis using continuous bead milling process for recovery of COD as model enzyme.

## Materials and Methods

### Microbial Strain and Growth Conditions

All the biochemical, chemicals, and bacterial cultivation media or their components were procured from RFCL (India), Hi-Media Laboratories (India), and BDH (India). Microbial culture conditions and COD expression studies were followed as reported earlier (Haque et al., 2016). COD expression studies were conducted using *E. coli* BL21 (DE3) pLysS cells (Invitrogen), transformed pFT24b+ harboring COD gene, under IPTG inducible lambda phage promoter (Invitrogen) following the protocol of Volontè et al. (2010). A single colony was used to initiate starter cultures in *E. coli* in Luria Bertani (LB) medium containing 30 mg mL^-1^ of kanamycin. Baﬄed Erlenmeyer flasks (500 ml) with 80 ml of the culture medium was inoculated and incubated at 37°C in a rotary orbital shaker at 180 rpm till the OD_600__nm_ reached 0.6. The cells were primarily grown in LB medium and then transferred to fermenter level in Terrific Broth (TB) supplemented with 12 g L^-1^ bacto-tryptone, 24 g L^-1^ yeast extract, 8 ml L^-1^ glycerol, 16 mM KH_2_PO_4_, and 54 mM K_2_HPO_4_. The cells were induced for protein expression after 6 h of incubation and harvested after 6 h of induction.

### Batch Culture

TB medium supplemented with 20 mL L^-1^ glycerol was used as a production medium in a 5 L glass bioreactor (Biostat^®^ C, Sartorius AG, Germany). The fermentation process was conducted at 37°C with 300–700 rpm agitation speed. The aeration rate was maintained at 1 vvm (volume of air per volume of medium per minute) and cascaded with pure oxygen. Hodag antifoam was used to control foaming *via* an antifoam sensor. An overnight culture of 0.6 (OD_600__nm_) grown in LB medium was used as inoculum and added at 10% (v/v) to the fermenter. The culture was induced with IPTG at a final concentration of 1.0 mM, at 6 h of incubation for the production of recombinant COD. All the experiments were performed in triplicates in two sets throughout this study.

### Determination of Enzyme Activity

The activity of COD was determined by evaluating the amount of H_2_O_2_ released by using 4 mg mL^-1^ horseradish peroxidase assay (with 0.3 mg mL^-1^ *o*-dianisidine, Δ𝜀440 = 13 mM^-1^ cm^-1^) at 25°C as per previous reports published (Pollegioni et al., 1999; Motteran et al., 2001). The reaction mixture contained 1 mM cholesterol in 100 mM phosphate buffer (pH 7.5) in the presence of 1% propanol and 1% Theist^®^ (v/v final concentration). One unit of the enzyme was defined as the amount of enzyme that produces 1 μmol of H_2_O_2_ min^-1^ at 25°C. Recovered lysate was centrifuged at 10,000 rpm and remaining supernatant was used for calculating the enzyme activity.

### Cell Lysis Using Ultra-Sonication

The obtained culture was subjected to centrifugation and the obtained pellet was washed twice with Tris–HCl buffer (pH 7.5) and resuspended in the same buffer. The cell lysis process was performed as reported earlier (Haque et al., 2016).

### Cell Lysis Using Bead-Mill

Bead mill known as Dynomill^®^ KDL type from W.A. Bachofen AG, Switzerland was used to lyse the cells. The grinding chamber (600 mL capacity) was filled with 0.50–0.75 mm glass beads in different amounts (v/v) as determined by the design matrix generated by RSM (**Table 1**). The chamber was cooled to 4°C for 10 min using chilled water from a circulating water bath. The cell slurry was adjusted at various cell densities and loaded in the grinding chamber to make up the remaining volume (as depicted in **Table 1**). The mill was run at 4000 rpm for 30 min or as directed by the design matrix. The cell slurry was fed in the grinding chamber at various flow rates with the help of pre-calibrated peristaltic pump. The bead mill was operated in continuous mode with slurry fed and withdrawn at equal flow rates.

**Table 1:** Design of experiments and recovered cholesterol oxidase (g L^-1^) in each run.

Run order	A: Feed rate (mL^-1^h)	B: Bead loading (%)	C: Cell loading (OD_600__nm_)	D: Run time (min)	Experimentally observed COD (g)	RSM predicted COD (g)	ANN predicted COD (g)
1	300.00	60.00	50.00	20.00	1.76	1.69	1.752
2	500.00	60.00	50.00	20.00	0.79	0.81	0.774
3	300.00	80.00	50.00	20.00	1.46	1.34	1.417
4	500.00	80.00	50.00	20.00	0.84	0.76	0.596
5	300.00	60.00	70.00	20.00	2.51	2.49	2.120
6	500.00	60.00	70.00	20.00	0.85	0.83	0.848
7	300.00	80.00	70.00	20.00	2.26	2.16	2.294
8	500.00	80.00	70.00	20.00	0.82	0.79	0.749
9	300.00	60.00	50.00	30.00	1.95	1.92	2.142
10	500.00	60.00	50.00	30.00	1.07	1.05	1.218
11	300.00	80.00	50.00	30.00	2.27	2.17	2.209
12	500.00	80.00	50.00	30.00	1.63	1.59	1.706
13	300.00	60.00	70.00	30.00	2.97	2.94	2.208
14	500.00	60.00	70.00	30.00	1.23	1.29	0.936
15	300.00	80.00	70.00	30.00	3.28	3.2	3.023
16	500.00	80.00	70.00	30.00	1.88	1.83	1.700
17	258.58	70.00	60.00	25.00	2.15	2.46	2.228
18	541.42	70.00	60.00	25.00	0.84	0.88	0.766
19	400.00	55.86	60.00	25.00	1.84	1.84	1.750
20	400.00	84.14	60.00	25.00	1.64	1.99	2.016
21	400.00	70.00	45.86	25.00	1.73	1.96	1.705
22	400.00	70.00	74.14	25.00	2.58	2.7	2.082
23	400.00	70.00	60.00	17.93	1.26	1.48	1.346
24	400.00	70.00	60.00	32.07	2.26	2.38	2.212
25	400.00	70.00	60.00	25.00	2.28	2.24	2.169
26	400.00	70.00	60.00	25.00	2.77	2.24	2.169
27	400.00	70.00	60.00	25.00	2.23	2.24	2.169
28	400.00	70.00	60.00	25.00	2.20	2.24	2.169
29	400.00	70.00	60.00	25.00	2.36	2.24	2.169
30	400.00	70.00	60.00	25.00	2.30	2.24	2.169

### Modeling and Optimization of Cell Lysis for COD Recovery

Each row in central composite design (CCD), **Table 1** represents an experimental run. Feed rate (A), bead loading (B), cell loading (C), and run time (D) were found to be significant process variables (Haque et al., 2016) and therefore were varied as per the values assigned by CCD. CCD, a subgroup of RSM was used in full factorial mode to form the basis of ANN and GA modeling. The process variables were varied at five levels (-2, -1, 0, +1, and +2) with constrains as shown in **Table 1**. The full factorial model resulted in a total of 30 runs. Each run was performed in triplicates (30 runs × 3 = 90 runs) and the COD recovery was quantified twice from each run. The COD recoveries thus obtained were averaged and fed into the design matrix against each individual run. *F*-test, analysis of variance (ANOVA) were performed and statistical significance of the model was estimated.

The results from the CCD experiment were used to train a feed-forward back-propagation ANN on MATLAB^®^ (MathWorks, Inc.) platform. Inputs and targets of the network were normalized. The four determinants of COD recovery (A, B, C, and D; **Table 1**) served as network inputs. The observed relative area of the dimer peak and shelf life (**Table 1**) served as training targets of the network. From the total 30 experiments (**Table 1**), 20 were selected and used to train the network while the rest were divided equally to test and validate it. The network was trained with the following parameters (training function, Levenberg–Marquardt Levenberg, 1944), Marquardt (1963); learning function, gradient descent with momentum weight and bias (Snyman, 2005); performance function, mean squared error. After optimization, ANN was also used to draw plots, illustrating the effect of individual components on COD recovery. The GA input parameters have been given in Electronic Supplementary Information (ESI), Annexure I. Since the GA implementation of MATLAB^®^ is designed to minimize, the output of the selection function (@beads) was made negative by multiplying by -1. Kindly refer (ESI): Annexure I for detailed GA option set.

## Results

### Bacterial Culture and COD Recovery

Expression of COD was successfully achieved at the shake flask and fermenter levels as reported earlier (Motteran et al., 2001; Haque et al., 2016). The expression was checked in the extracellular medium after centrifuging the cells at 10,000 rpm. The supernatant showed no COD activity confirming its expression to be completely intracellular. The amount of COD expressed per unit cell mass (OD_600__nm_) mL^-1^ was ~5.89 μg. The estimation was done by determining the activity of crude COD in the cell lysate. Sodium dodecyl sulphate-polyacrylamide gel electrophoresis (SDS-PAGE) was run to confirm the expression of COD (data not shown). The molecular weight of COD was estimated to be ~46.5 kDa (Haque et al., 2016).

### Evaluation of Cell Lysis

The *E. coli* cells were lysed to estimate the maximum possible recovery of COD per unit cell mass. The cell slurry was diluted to OD_600__nm_ = 10, and sonicated for 25 min (15 s “ON”/30 s “OFF” cycle) in an ice bath. The recovered amount was ~5.89 μg COD mL^-1^ OD_600nm^-1^_, with the maximum COD activity of ~91.42 units. The recovery (COD) results were consistent with our previous study (Haque et al., 2016). The recovery of 5.89 μg COD mL^-1^ OD_600nm^-1^_ was considered as 100% and utilized for further yield and recovery calculations.

### Bead Size and Bead Milling Time

Bead size of 0.5–75 mm was selected for optimization process and bead milling time of 30 min was utilized to build the ANN design. The selection criteria have already been reported in our previous report (Haque et al., 2016).

### Design of Experiments Using RSM

CCD, as subset of RSM was utilized for statistical design and modeling. Full factorial design matrix was utilized that resulted in to a set of 30 runs, with simultaneous variation across all the participating parameters at five different levels as depicted in **Table 1**. Each run was executed in triplicate and repeated twice. The recovered amount of COD was averaged and inserted to the design matrix against individual runs. The minimum amount of COD recovered was 0.794 gL^-1^ in run 2 and the maximum amount of COD obtained was 3.282 gL^-1^ in run 15. Maximum response was 4.133 times of the minimum. The model showed a *F*-value of 16.8, indicating that this high *F*-value is relevant and the chances that *F*-value this high can occur due to noise, is very small. The model showed 9 degrees of freedom (df) for “lack of fit” and 5 df for pure error. The individual terms in the model as A, C, and D were significant terms and AC, BD, A^2^, B^2^, and D^2^ were found to be significant interaction terms with *p*-values < 0.05 for individual and <0.1 for interaction terms. Individual terms B, and interaction terms AB, AD, BC, CD, and C^2^ were not found to support the model hierarchy or show significance (*p*-value >0.1), therefore, these terms were excluded from the RSM model for better curve fitting and maintaining the simplicity. ANOVA for bead loading was not found significant (*F*-value = 0.3213) and therefore, it was excluded from the response surface model. We know that that grinding of the cells happens due to the presence of beads; and bead fraction present in the grinding chamber is directly proportional to the fraction of the lysed cells. The *F*-value for feed rate, run time, and cell loading were found to be 124, 40.34, and 27.13, respectively, suggested that these parameters have profound effect on the process of cell lysis for COD recovery. Bead loading showed a lower *F*-value of 1.06 indicating its lower significance in the complete process. The degree of precision, as shown by CV at 12.05%, indicated that the developed model was acceptable and sustainable for generating the prediction functions for COD recovery at different design points throughout the design space. Adequate precision that confers signal-to-noise ratio was 14.859. The coefficient of determination was found to be 0.9474 with predicted *R*^2^ in reasonable agreement with adjusted *R*^2^ confirming the aptness and reliability of the model (**Table 2**). As per the statistical standards, the value of coefficient of determination should be 0.95 or higher for an accurate curve fit. Therefore, the prediction accuracy of the RSM model reduced below the 95% confidence levels. The formulated polynomial equation in coded terms, to navigate the design space can be shown as:

**Table 2:** ANOVA for cholesterol oxidase recovery (g L^-1^).

	ANOVA for Response Surface Reduced Cubic Model					
	Analysis of variance table (partial sum of squares—Type III)					
Source	Sum of squares	df	Mean square	*F*-value	*p*-value Prob > *F*	
Model	12.76	15	0.85	16.8	<0.0001	Significant
A: Feed rate	6.28	1	6.28	124	<0.0001	
B: Bead loading	0.054	1	0.054	1.06	0.3213	
C: Cell loading	1.37	1	1.37	27.13	0.0001	
D: Run time	2.04	1	2.04	40.34	<0.0001	
AB	0.084	1	0.084	1.65	0.2199	
AC	0.62	1	0.62	12.21	0.0036	
AD	4.23E-05	1	4.23E-05	8.34E-04	0.9774	
BC	2.10E-04	1	2.10E-04	4.15E-03	0.9495	
BD	0.35	1	0.35	6.88	0.0200	
CD	0.048	1	0.048	0.95	0.3470	
A^2^	0.76	1	0.76	14.99	0.0017	
B^2^	0.25	1	0.25	4.94	0.0432	
C^2^	0.018	1	0.018	0.35	0.5621	
D^2^	0.23	1	0.23	4.47	0.0529	
ABC	5.63E-05	1	5.63E-05	1.11E-03	0.9739	
Residual	0.71	14	0.051			
Lack of fit	0.48	9	0.054	1.2	0.4437	Not significant
Pure error	0.22	5	0.045			
Cor total	13.47	29				
Standard deviation	0.23		*R*^2^	0.9474		
Mean	1.87		Adjusted *R*^2^	0.891		
Coefficient of Variation%	12.05		Predicted *R*^2^	0.8137		
PRESS	2.51		Adequate precision	14.859		

$$\begin{array}{ccc} Y & = & +2.22-0.57\times A+0.077\times B+0.26\times C\\ \; & \; & +0.32\times D+0.072\times A\times B-0.20\times A\times C\\ \; & \; & +0.15\times B\times D+0.055\times C\times D-0.26\times A^{2}\\ \; & \; & -0.094\times B^{2}-0.17\times D^{2} \end{array}$$

**FIGURE 1:**
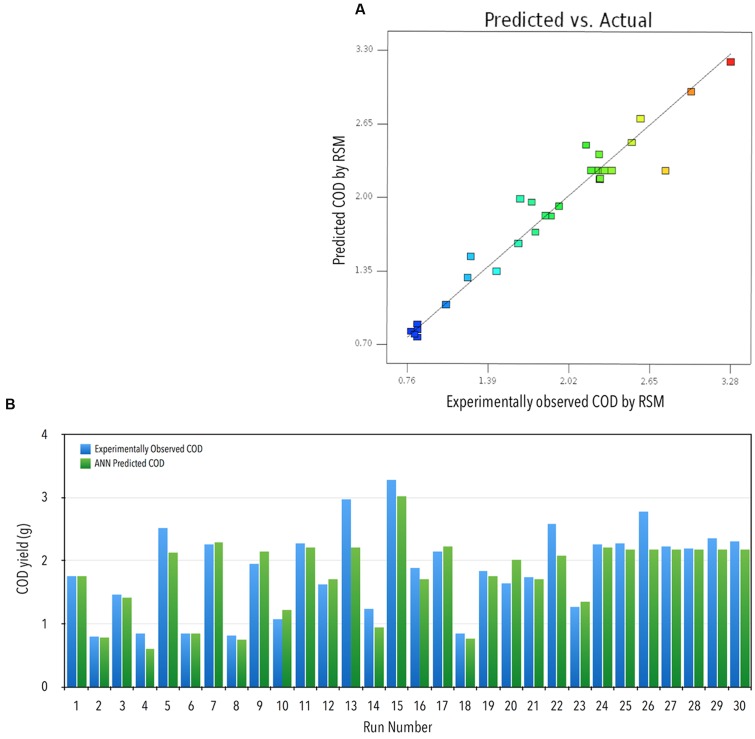
**(A)** Actual vs. RSM predicted COD yield. **(B)** Comparison of observed and ANN predicted COD recovery (g): experimentally observed COD yield vs. ANN predicted COD yield against various runs.

**FIGURE 2:**
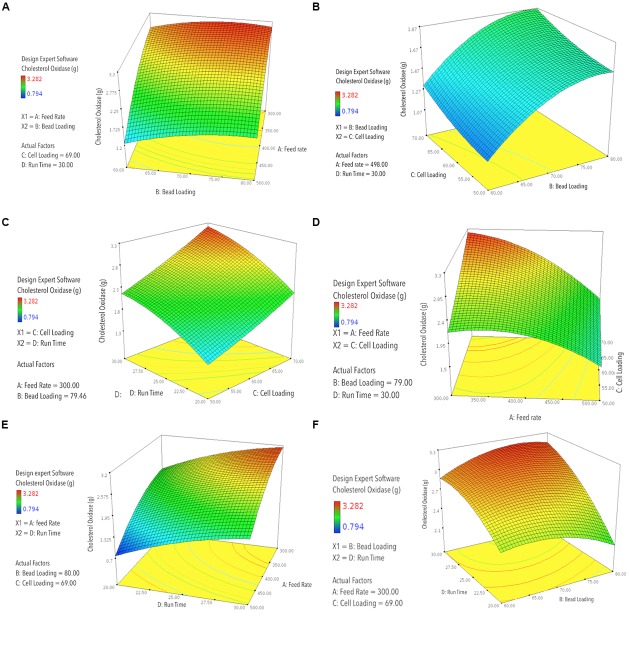
**RSM-3D contour graphs plotted for two participating factors at one time, on *x*- and *y*-axis, against the obtained responses for COD recovery plotted on the *z*-axis, with different levels indicated by the color/height/contours of the obtained curve; (A)** predicted COD recovery by RSM against feed rate (mL h^-1^) vs. bead loading (%, v/v); **(B)** predicted COD recovery by RSM against bead loading (%, v/v) vs. cell loading (OD_600__nm_); **(C)** predicted COD recovery by RSM against cell loading (OD_600__nm_) vs. run time (min); **(D)** predicted COD recovery by RSM against feed rate (mL h^-1^) vs. cell loading (OD_600__nm_); **(E)** predicted COD recovery by RSM against feed rate vs. run time (min); **(F)** predicted COD recovery by RSM against bead loading (%, v/v) vs. run time (min).

Where, *Y* = COD recovered (g L^-1^), *A* = feed rate (mL/h), *B* = bead loading (%, v/v), *C* = cell loading (OD_600__nm_), and *D* = run time (min), and the interacting factors were depicted as AB, AC, BD, and CD. The developed model showed good correlation between the predicted and the observed responses, ensuring a good fit between the two (**Figure 1A**). The 3D graphs plotted using the two participating factors at one time, on *x*- and *y*-axis, while the obtained responses for COD recovery was plotted on the *z*-axis, with different levels indicated by the color/height/contours of the obtained curve. These curves provide an easy and quick understanding of the responses predicted (**Figures 2A–F**). Validation of the developed model was done by performing the bead milling process at the predicted optimum condition in triplicate.

### Optimization Using ANN and GA

The DOE is represented in **Table 1**. Each row of **Table 1** is an individual combination of parameters affecting recovery of COD. The recovery potential for each individual run is present in column 6 of **Table 1**. The predictions for the tested and validated ANN are shown in column 7 of **Table 1** (**Figure 1B**).

**FIGURE 3:**
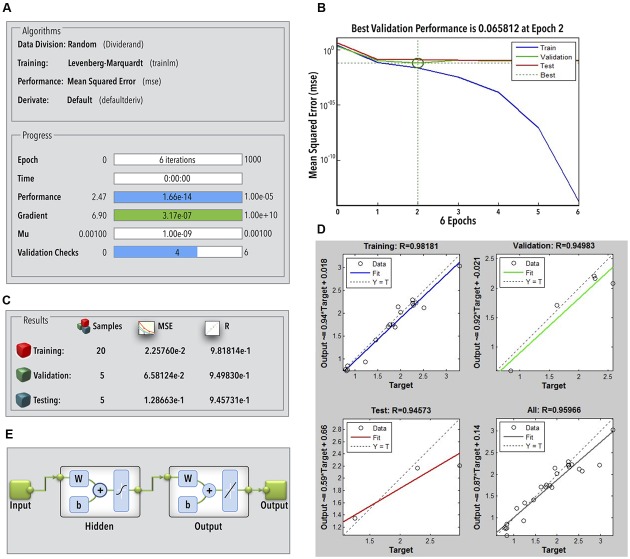
**(A)** ANN parameters during training; **(B)** ANN performance during training, validation, and testing; **(C)** statistical quality of the ANN; **(D)** *R*-value of ANN training, testing, and validation; **(E)** ANN structure (W, layer weights; b, layer bias).

**FIGURE 4:**
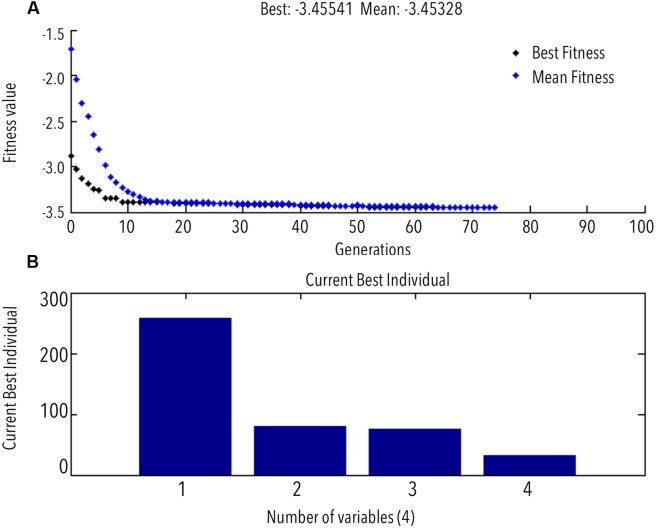
**(A)** GA predicted optimum (GA progress toward maximum COD recovery); **(B)** best/optimum combination of variables for maximum COD recovery.

The ANN achieved an acceptable degree of efficiency (mean validation error 0.065) after six iterations in two epochs only (**Figures 3A,B**). Individual *R*-values for training and validation are shown in **Figures 3C,D**. The regression coefficients for training, validation, and testing were fairly close to 1 (**Figure 3C**). This classifies the ANN to be fairly efficient to represent the relationship between the bead milling parameters (i.e., feed rate, bead loading, cell loading, and run time) and COD recovery. The number of hidden layers required to achieve the said efficiency of the ANN was optimized during multiple training and testing attempts. **Figure 3E** represents the final network in MATLAB^®^ Notation. Kindly refer ESI: Annexure I for network layer weights and biases. GA was employed to perform an exhaustive global search of the parameter combination to achieve maximum 3-COD recovery. The results of GA optimization as per the GA option-set (ESI: Annexure I) have been shown in **Figures 4A,B**.

GA predicted the best fitness value or the *in silico* maximum of COD recovery to be 3.454 g L^-1^ (**Figure 4**, negative sign explained in Section “Materials and Methods”) at an optimum feed rate (mL h^-1^): 258.08, bead loading (% v/v): 80%, cell loading (OD_600__nm_): 73.99, and run time of 32 min. This optimum combination of the solvent system components was tested experimentally in triplicate; the COD recovery was found to be within 1.5% of the GA predicted maximum.

**FIGURE 5:**
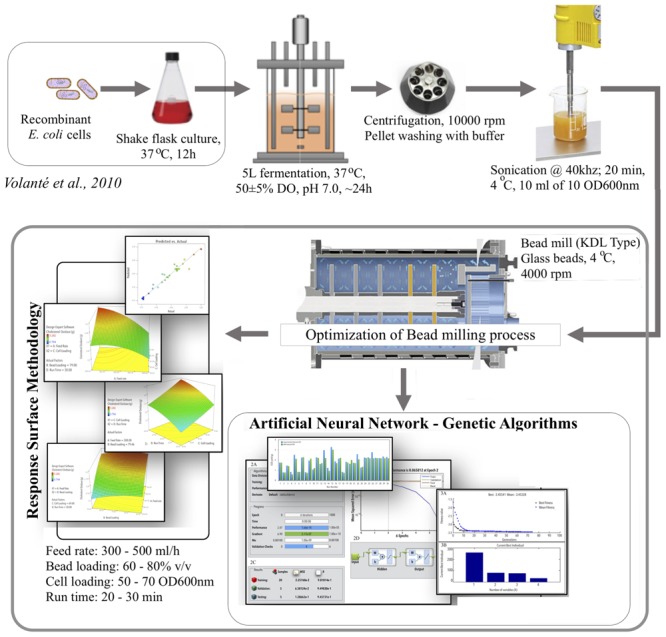
**Effect of various bead milling parameters on COD recovery: (A)** bead load (%) vs. COD yield (g); **(B)** run time vs. COD yield (g); **(C)** cell loading vs. COD yield (g); **(D)** feed rate vs. COD yield (g).

The validated ANN was also used to predict the individual effect of the milling parameters on the COD yield (**Figures 5A–D**). This effect was determined individually for each parameter separately by simulating the validated network for four sets of inputs each comprised of a four milling parameters, wherein only one is varied within its respective range, while keeping the rest three at the central values of CCD experiment (**Table 1**). Three of the four milling parameters, specifically, bead load, run time, and cell loading were found to have a positive hyperbolic effect on COD yield (**Figures 5A–C**). The feed rate was found to be positively related to COD yield for values below 350 mL h^-1^, a further increase in the feed rate results in a sharp decrease in COD yield (**Figure 5D**).

## Discussion

The production of intracellular recombinant proteins in *E. coli* offers ease of cloning and expression of foreign proteins and it can grow up to higher cell densities (Jawed, 2008) reaching close to ~100 OD_600__nm_; making the process highly productive for potential commercial applications. Once successfully expressed, the heterologous protein(s) needs to be extracted out of the cells and purified. The recovery of intracellularly expressed protein depends on many factors including the process of bacterial cell lysis. Laboratory scale cell lysis processes, e.g., ultra-sonication is easy and less cumbersome but loses its efficiency when the cell slurry is concentrated or higher in volume. Slow speed, low productivity, and thermal denaturation pose a big challenge. Chemical cell lysis process is fairly simple to perform but tend to be harsh, expensive, increases the burden of waste disposal and eﬄuent treatment and can interfere in downstream processing of the expressed protein. In both the methods, the protein expressed is prone to thermal or chemical denaturation. These limitations discourage their use at commercial levels. Cell lysis at industrial scale requires faster, efficient, and robust process capable of handling large volumes of cell slurry at high densities and viscosities. Bead milling process operates by the principle of grinding the cells between two beads that transfer their kinetic energy to break the cell continuum. Only scanty studies have documented the effect of various bead milling parameters, and hardly any study has accounted for their interaction-effects on protein recovery and overall productivity. Cell lysis (e.g., yeast cells) and protein recovery in a bead milling process follow a first order kinetics, when single process parameter is taken into consideration (Chisti and Moo-Young, 1986). This can be shown by the equation:

$$In\left(\frac{R_{m}}{R_{m}-R}\right)=In\left(\frac{1}{1-R_{p}}\right)=kt$$

Where, *R_m_* is the maximum weight of protein released per unit weight of packed cells (yeast) and *R* is the weight of actual protein recovered, *Rp* is the fractional protein released, *k* is the rate constant, and *t* is the time. When more than one parameter and their effect are studied simultaneously on the process, it becomes more intricate and necessitates equations of higher order for explaining the cell disruption kinetics. Increase in grinding time increases the residence time of every cell inside the grinding chamber, reducing the chances of any cell escaping impaction by the beads. Slurry feed rate on the other hand pushes fresh cells inside the grinding chamber, countering the process efficiency. Therefore, a dynamic balance is necessary between the feed rate and run time to achieve higher cell lysis. As the grinding requires a lot of power to drive the battery of beads in the grinding chamber *via* agitators; a slower feed rate would make the process run unnecessarily longer, compromising process economics; while a higher feed rate tends to reduce the residence time, affecting the protein. Increase in the amount of cells per unit volume also increase the yield of the process steadily as higher amount of the cells also harbor more intracellular content that is available for the release by cell disruption. As the amount of the cells increases, the total protein content as well as the viscosity of the cell slurry increases in the grinding chamber. The increase in the protein content is beneficial for the process, while the increase in the viscosity is generally detrimental. Moreover, as the cell lysis proceeds, the intracellular contents, e.g., nucleic acids such as DNA, RNA; enzymes, e.g., proteases, etc., are also spilled out of the cells along with the protein of interest. This worsens the situation as nucleic acids further increase the viscosity of the lysate and proteases threaten the degradation of the protein of interest. Increase in the viscosity requires the agitator drive to draw more power, limits grinding by reducing the bead velocity and the force of impaction. To overcome this limitation, a higher percentage of the beads are required to be loaded in the grinding chamber. Increase in the bead loading percentage accelerates the cell lysis, but generates more heat leaves less room for the cell slurry in the grinding chamber, reducing the productivity per unit time. Increase in the amount of the beads also generates more heat due to higher frequency of collision. As the bead loading increases, its agitation requires more power to drive the battery of beads against the gravity and the viscosity of the slurry inside. This results in higher power to be delivered to the agitator for maintaining the agitation speed and overcoming the inertia and friction of the beads continuously. Increasing the diameter of bead mill, limits the efficiency of cooling jacket that is along the circumference of the bead mill, making cooling less effective and result in generation of temperature gradient inside the bead mill. To overcome the limitation, bead size (0.25–1.0 mm) was optimized (Volontè et al., 2010). Smaller beads are effective at lower viscosities, but these gets fluidized when run at higher cell loads, higher viscosities or at high agitation speeds (Middleberg, 1995). Thus results in lower momentum of impaction, incomplete cell lysis and affects the process productivity. Larger bead sizes 0.5–0.75 mm prove effective as these provide stronger impact, deliver higher kinetic energy during impaction resulting in resilient shearing action leading to effective cell lysis at higher cell loads and viscosities. To further increase the effectiveness, the specific gravity of the beads employed can be increased. Operation of the bead milling process is fairly simple and straight-forward, its cell disruption kinetics is not. Continuous bead milling processes with slurry feeding are even more complex than the batch bead milling processes. A number of factors affect the productivity and the yield in more than one ways and therefore, simultaneous optimization of the process variables is required for accurate optimization of the continuous bead milling process.

RSM though capable of modeling an experimental study with simple interactions, finds itself limited when the process interactions grow complex. With a full factorial design (~30 runs) the number of design points increase (w.r.t fractional factorial design ~21 runs) and hence the data generated is more elaborate. The ANOVA for RSM demonstrated bead loading and many important terms, e.g., B, AB, AD, BC, CD, C^2^, and ABC to be a non-significant parameter; however, we know that it is a very crucial parameter for effective cell lysis. Still the RSM model was able to fit the observed responses into a statistically significant model, with a not-significant “lack-of-fit,” acceptable predictability, but with lower a confidence level <95%. In the study undertaken, the feed rate and bead loading have a very complex effect on the COD recovery. Increase in the feed rate reduces the residence time of each cell in the grinding chamber, making it less prone to lysis; increase in the bead loading percentage increases the amount of grinding elements in the chamber making the process more effective. On the other hand, increase in bead loading reduces the available volume for the cell slurry in the grinding chamber, boosting the flow rate and progressively reducing the residence time of the cells in the grinding chamber. This makes the interaction too complex for RSM to accurately predict the response. Similarly, the interaction of feed rate and cell loading compensates the effect of each other. Higher flow rate reduces the efficiency while higher cell loading increases the number of cells per unit volume to be lysed. As the cell concentration increases, the efficiency of cell lysis decreases due to increase in the viscosity. This generates the need for more beads to be loaded in the grinding chamber. Interaction of feed rate (A), bead loading (B), and cell loading (C) affect the run time (D) in a complex manner thereby affecting the recovery of COD. ANNs are effective in handling the data with noise, mimic biological neural networks, perform better than statistical/regression based models and accurately predict and model highly non-linear and complex biological processes. The present study highlights the effectiveness of machine learning algorithms (*viz.* ANN) over statistical approach, in explaining the complex dependence of the desired product recovery on industrial downstream variables (in this case, bead milling parameters). Results (as discussed before) clearly identify the advantage of using ANN as compared to regression based statistical methods such as RSM.

The bead mill has a lot of advantages over laboratory scale cell lysis methods, but still it is not free of drawbacks. The bead mill can be operated in batch or continuous mode. After every batch run, the grinding chamber and used beads need to be cleaned, sanitized, and dried, in order to be used in the next run. This downtime and its cost gets added to volume of the cell slurry processed every time, negatively affecting process economics. With every cleaning cycle a fraction of grinding beads is lost depending on the carefulness and the skill of the operator. To increase the processing volume, larger bead mills are needed that makes them bulky, cumbersome, and inefficient. Therefore, smaller bead mills with provision of continuous feeding of fresh cell slurry are employed. Fresh slurry is continuously fed in the grinding chamber and an equivalent amount is collected from the outlet valve. The gap setting of the outlet valve retains the beads while allows the cell slurry to flow-out due to the inlet pressure. Continuous bead milling process reduces the downtime as it handles larger volumes than the grinding chamber, needs cleaning and sanitization only after the complete slurry is processed. Therefore, the downtime is reduced and the volumetric productivity is increased per grinding cycle. A small in-house economic analysis was conducted to compare the process economics in the case of methods optimized by statistical techniques and ANN-GA. The in-house economic analysis revealed that optimization by ANN-GA resulted in 3.7-folds increase in the amount of COD recovered per USD per day as compared to only RSM optimization under batch mode (Annexure II). In comparison with the bead milling under batch mode (6.56 mg COD/USD/day), ANN-GA resulted into 24.33 mg COD/USD/day).

## Conclusion

In conclusion, the present study proves the significance of ANN-GA in large-scale bacterial cell lysis and product recovery (COD in this case) in optimization of bead milling process for the very first time. It also refines and establishes the crucial parameters and their interactions affecting the cell lysis and recovery of intracellularly expressed proteins. The COD recovery reached up to 3.454 g L^-1^ as compared to the batch or the continuous cell lysis process optimized by RSM. Our findings provide a concrete platform for the large-scale cell lysis process and enhanced recovery of intracellular protein(s) in a continuous bead milling process with expressions depicting similar patterns.

## Author Contributions

AJ and SK conceived and designed the experiments. SH, VS, MW, and SD performed the experiments. AJ, SH, SK, RM, and DT analyzed the data. NS, MA, TG, and HK contributed reagents/materials/analysis tools/proofing. AJ, SK, SH, and ML wrote the paper.

## Conflict of Interest Statement

The authors declare that the research was conducted in the absence of any commercial or financial relationships that could be construed as a potential conflict of interest. The reviewer FG and handling Editor declared their shared affiliation, and the handling Editor states that the process nevertheless met the standards of a fair and objective review.
