# Supplementary material for: Artificial Intelligence vs. Statistical Modeling and Optimization of Continuous Bead Milling Process for Bacterial Cell Lysis
Source: Front Microbiol. 2016 Nov 22;7:1852. doi: 10.3389/fmicb.2016.01852 (PMC5118707; doi:10.3389/fmicb.2016.01852)
Supplement: Supplementary file 1 [file Data_Sheet_1.DOCX]

***Annexure I***

**ANN**

**Input Weights**

0.455887200717575 -1.49961689112863 0.888368516611610 1.70721150166497

1.72339595934045 0.691177820347992 1.47592924036038 -0.855744844638386

-2.44494459114790 1.10866687619402 0.186356614838822 1.34456984579341

2.07588372686005 1.08233558795723 0.632225509518742 0.744601306731205

1.49902406332118 0.907942636588448 1.73558453554780 -0.815386152096850

0.788595652344552 -0.714584493421162 -0.332090933400412 -1.86475900588171

-0.408031580569097 -0.266244938478423 -1.78942439443842 -1.64189466096356

-1.09965663049731 0.823876644955006 2.28184095591627 0.128608306825077

1.29002554631095 -1.45183262359824 -0.192075981089585 -1.19554743999038

-1.36389981892962 1.49431876520163 -1.38479721234471 0.642535342739855

**Layer Weights**

-0.209969463339944 -0.162293101074810 0.422222247321430 -0.232811336170303 0.0318983787823972 -0.0599049844600295 -0.220613062454729 -0.0214218496131801 -0.592060063358069 -0.330204384584667

**Bias**

**Input bias**

-2.44531535767571

-1.94061109799133

1.31020308411927

-0.922383209576274

-1.05777135527613

0.551048585352175

-0.707662491329896

-1.01546069026471

2.86286625830053

-2.35845522256314

**Output bias**

-0.623376115449628

**GA Input Parameters**

Following are the GA input parameters (crossover fraction, 1; elite count, 2; population size, 200; migration direction, forward; migration interval, 20; migration fraction, 0.2; generations 150; stall gen limit, 50; creation Fcn, @gacreationuniform; fitness scaling Fcn, rank wise; gaproblem.fitnessfcn:@beads used in the MATLAB^®^ implementation of GA for optimization.

**GAoptions = Default otherwise mentioned**

PopulationType: []

PopInitRange: []

PopulationSize: 200

EliteCount: []

CrossoverFraction: []

ParetoFraction: []

MigrationDirection: []

MigrationInterval: []

MigrationFraction: []

Generations: 50

TimeLimit: []

FitnessLimit: []

StallGenLimit: 10

StallTimeLimit: []

TolFun: []

TolCon: []

InitialPopulation: [200x3 double]

InitialScores: [200x1 double]

InitialPenalty: []

PenaltyFactor: []

PlotInterval: []

CreationFcn: []

FitnessScalingFcn: []

SelectionFcn: []

CrossoverFcn: @crossoverscattered

MutationFcn: []

DistanceMeasureFcn: []

HybridFcn: []

Display: 'off'

PlotFcns: {[@gaplotbestf] [@gaplotbestindiv]}

OutputFcns: []

Vectorized: []

UseParallel: []

**All the [] options are at default values.**
