# Supplementary material for: Artificial Intelligence vs. Statistical Modeling and Optimization of Continuous Bead Milling Process for Bacterial Cell Lysis
Source: Front Microbiol. 2016 Nov 22;7:1852. doi: 10.3389/fmicb.2016.01852 (PMC5118707; doi:10.3389/fmicb.2016.01852)
Supplement: Supplementary file 2 [file Data_Sheet_2.DOCX]

| Economic analysis of Bead milling process for bacterial lysis (e.g. COD recovery) | | | | | | | |
| --- | --- | --- | --- | --- | --- | --- | --- |
| Steps in Bead milling process | | **Batch Cell Lysis Process (274 ml) – Reference No. 18 in Manuscript** | | | **Continuous Cell Lysis Process (264 ml *4 = 1056 ml) – Current Study** | | |
|  |  | Description | Requirement | Approx. Cost (INR)* | Description | Requirement | *Approx. Cost (INR) |
| Preparation and assembly | Step 1: Washing and assembly | Manpower required | 1 day | 1200.00 | Manpower required | 1 day | 1200.00 |
|  | Step 2: Measuring and filling of beads | Glass beads required | 465 ml | -- | Glass beads required | 480 ml | -- |
|  | Step 3: Preparation of cell slurry & filling of grinding chamber | Tris-HCL Buffer | 1 L | 794.00 | Tris-HCL Buffer | 1 L | 794.00 |
|  | Step 4: Initiation of cooling water bath | Cooling (2kWh) | 30 min | 10.00 | Cooling (2kWh) | 30 min | 10.00 |
|  |  | **(A) Preparation cost** |  | **2004.00** | **(A) Preparation cost** |  | **2004.00** |
| Running of bead mill | Step 5: Running of bead mill, | Electricity for Milling (1.8kWh) | 30 min | 9.00 | Electricity for Milling (1.8kWh) | 160 min | 48.00 |
|  | Feeding of cell slurry (only in continuous mode) | Electricity for cooling (2kWh) | 30 min | 10.00 | Electricity for cooling (2kWh) | 160 min | 54.00 |
|  | Step 6: Collection of cell lysate | Collection of cell lysate | Glass tray | Misc. | Collection of cell lysate | Glass tray | Misc. |
|  |  | **(B) Running cost** |  | **19.00** | **(B) Running cost** |  | **102.00** |
| Cleaning / Sanitization | Step 7: Bead removal (decantation / sedimentation) | Removal of beads | Glass tray | Misc. | Removal of beads | Glass tray | Misc. |
|  | Step 8: Cleaning / Sanitization | Water for cleaning of beads | 10 L | 6450.00 | Water for cleaning of beads | 10 L | 6450.00 |
|  | Step 9: Drying of beads at 80^o^C in oven | Sodium Hydroxide for Sanitization | 1 L | 1229.00 | Sodium Hydroxide for Sanitization | 1 L | 1229.00 |
|  |  | Drying of beads in Oven (2kWh) | 10h | 200.00 | Drying of beads in Oven (2kWh) | 10h | 200.00 |
|  |  | **(C) Cleaning/Sanitization** |  | **7879.00** | **(C) Cleaning/Sanitization** |  | **7879.00** |
|  |  | Total Volume of Slurry processed | 274 ml |  | Total Volume of Slurry processed | 1056 ml |  |
|  |  | Total COD recovered | 975.44 mg |  | Total COD recovered | 3647.42 mg |  |
|  |  | Total process time (approx.) | 11 h |  | Total process time (approx.) | 13 h |  |
|  |  | **Bead loss ( D )** | **~ 1 %** | **150.00** | **Bead loss ( D )** | **~ 1 %** | **150.00** |
|  |  | **Total cost (A+B+C+D)** |  | 10052.00 | **Total cost (A+B+C+D)** |  | 10135.00 |
|  |  | Total cost of running in USD** |  | 150.23 | Total cost of running in USD** |  | 151.47 |
|  |  | **Productivity (mg COD/USD/day)** |  | **6.56** | **Productivity (mg COD/USD/day)** |  | **24.33** |
|  |  | ***Fold Increase in COD Productivity Continuous vs. Batch cell lysis = 24.33/6.56 = 3.70*** | | | | | |

**Note:** The calculation doesn’t take into account the fixed costs (Bead mill, Glass beads, Cooling water bath, Feed pump, incubator. Misc. items include: Glass tray, measuring cylinders, tubing, beakers, funnel, etc).

* The prices shown here are indicative only and subject to change as per the country norms of local taxes, freight charges, VAT, etc., therefore, the final cost may not remain the same.

** Currency conversion 1 USD = 66.91 INR, as on Jun 12^th^, 2016.
